# A sequence of SVA retrotransposon insertions in *ASIP* shaped human pigmentation

**DOI:** 10.1038/s41588-024-01841-4

**Published:** 2024-07-24

**Authors:** Nolan Kamitaki, Margaux L. A. Hujoel, Ronen E. Mukamel, Edward Gebara, Steven A. McCarroll, Po-Ru Loh

**Affiliations:** 1 Division of Genetics, Department of Medicine, Brigham and Women’s Hospital and Harvard Medical School, Boston, MA USA; 2 Center for Data Sciences, Brigham and Women’s Hospital, Boston, MA USA; 3 Program in Medical and Population Genetics, Broad Institute of MIT and Harvard, Cambridge, MA USA; 4 Stanley Center for Psychiatric Research, Broad Institute of MIT and Harvard, Cambridge, MA USA; 5 Department of Genetics, Harvard Medical School, Boston, MA USA; 6 Department of Biomedical Informatics, Harvard Medical School, Boston, MA USA

**Keywords:** Genetic association study, Gene expression, Skin cancer, Population genetics

## Abstract

Retrotransposons comprise about 45% of the human genome^1^, but their contributions to human trait variation and evolution are only beginning to be explored^2,3^. Here, we find that a sequence of SVA retrotransposon insertions in an early intron of the *ASIP* (agouti signaling protein) gene has probably shaped human pigmentation several times. In the UK Biobank (*n* = 169,641), a recent 3.3-kb SVA insertion polymorphism associated strongly with lighter skin pigmentation (0.22 [0.21–0.23] s.d.; *P* = 2.8 × 10^−351^) and increased skin cancer risk (odds ratio = 1.23 [1.18–1.27]; *P* = 1.3 × 10^−28^), appearing to underlie one of the strongest common genetic influences on these phenotypes within European populations^4–6^. *ASIP* expression in skin displayed the same association pattern, with the SVA insertion allele exhibiting 2.2-fold (1.9–2.6) increased expression. This effect had an unusual apparent mechanism: an earlier, nonpolymorphic, human-specific SVA retrotransposon 3.9 kb upstream appeared to have caused *ASIP* hypofunction by nonproductive splicing, which the new (polymorphic) SVA insertion largely eliminated. Extended haplotype homozygosity indicated that the insertion allele has risen to allele frequencies up to 11% in European populations over the past several thousand years. These results indicate that a sequence of retrotransposon insertions contributed to a species-wide increase, then a local decrease, of human pigmentation.

## Abstract (web summary)

Genomic analysis identifies an SVA retrotransposon insertion in an intron of *ASIP* as a likely causal variant influencing human pigmentation. This insertion appears to mitigate the effects of an older, nonpolymorphic SVA insertion in the same intron.

## Main

Variation in skin pigmentation has profoundly influenced human evolution and social history, enabling *Homo sapiens* to adapt to environments with diverse levels of solar radiation. Agouti signaling protein (ASIP) is a secreted protein that plays a key role in skin and hair pigmentation by binding to a receptor (melanocortin 1 receptor (MC1R)) on the surface of melanocytes, causing them to shift melanin pigment production from darker, brown eumelanin to lighter, red pheomelanin^7^. Across vertebrates, regulated increases in expression of ASIP decrease pigmentation temporally and in different parts of the body^8^. In humans, the ASIP-MC1R pathway is affected by several of the largest influences of common genetic variation on skin and hair pigmentation, including several common missense variants in *MC1R*^9,10^.

Genome-wide association studies (GWAS) in European genetic-ancestry cohorts for pigmentation-related traits, including skin cancers such as melanoma, have long observed a particularly strong association near the *ASIP* gene^4–6^ that colocalizes with an expression quantitative trait locus (eQTL) for *ASIP*^11^. However, a plausible functional variant for this common, large effect (>0.2 s.d. change in pigmentation phenotypes) has not been identified, despite the considerable statistical resolution afforded by large biobank cohorts^12^. GWAS of African cohorts (from Ethiopia, Tanzania, Botswana or KhoeSan populations)^13,14^ or East Asian cohorts (from Japan or Korea)^15,16^ have not found an association at this locus, suggesting that the functional variant emerged recently and on a European-specific haplotype, consistent with a genome-wide scan of recent positive selection in a British cohort that identified the *ASIP* locus among other pigmentation-associated genes^17^.

Across mammals, structural mutation at *ASIP* is a recurring mechanism underlying variation in coat color. Changes in coat color have occurred by large rearrangements at *ASIP* in lethal yellow agouti mice (*A*^*y*^)^18^, sheep^19^ and gibbons^20^, and by retrotransposon insertions in viable yellow agouti mice (*A*^*vy*^)^21^ and dogs^22^. However, such polymorphisms have not been reported for human *ASIP*.

**Fig. 1:**
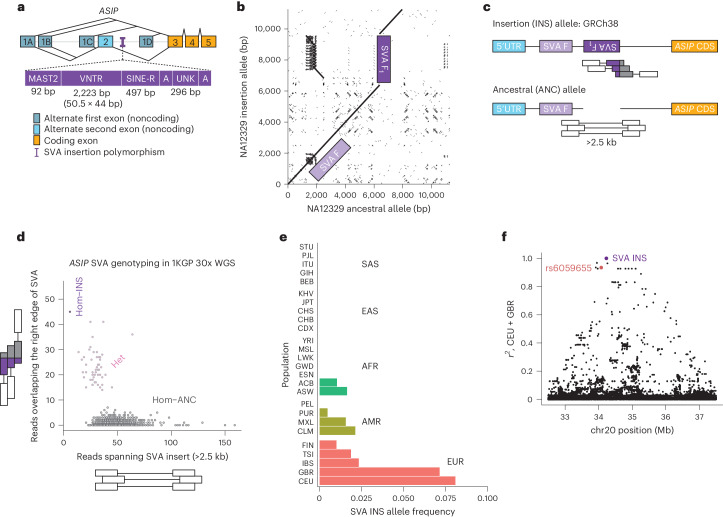
Characterization of a polymorphic SVA F_1_ retrotransposon insertion within an intron of *ASIP.* **a**, Architecture of *ASIP* isoforms and composition of the SVA F_1_ retrotransposon insertion. *ASIP* has four known alternate first exons (dark blue), with the first three (exons 1A, 1B and 1C) able to either splice directly to a coding exon (gold; exon 3) or first to an additional 5′ UTR exon (light blue; exon 2). The SVA F_1_ insertion contains the expected 5′ truncation with *MAST2* exon followed by VNTR and SINE-R sequences. The location of the SVA F_1_ insertion, present in the GRCh38 reference, is indicated by the purple thin vertical bar. **b**, Pairwise sequence alignment dotplot of long-read sequencing-derived haplotypes^23^ from an individual heterozygous for the SVA F_1_ insertion (NA12329). The SVA F_1_ insertion, present in the haplotype on the *y* axis, corresponds to the vertical break in the diagonal alignment (purple) and bears substantial homology (dashes to the left of the vertical break) to a nonpolymorphic SVA F retrotransposon 3.9 kb upstream (light purple). **c**, Genotyping approach for short-read data. Read alignments overlapping the right breakpoint of the SVA F_1_ indicate the presence of at least one insertion allele, whereas discordant read pairs with excessively long fragment sizes indicate the presence of at least one non-insertion allele (Methods). CDS, coding sequence. **d**, Determination of SVA insertion genotypes for individuals in the 1KGP. Individuals homozygous for the ancestral allele without the SVA F_1_ insertion (Hom-ANC; gray) are the most common and have no or few reads overlapping the right breakpoint; individuals homozygous for the insertion allele (Hom-INS; purple) are the least frequent and have few or no reads with fragment size >2.5 kb; heterozygous individuals have both read types (Het; pink). **e**, SVA F_1_ insertion allele frequency across 1KGP populations. The insertion is present in European genetic ancestry populations and others with known European admixture but is otherwise absent. **f**, Extensive LD (*r*^2^) of the SVA F_1_ insertion (at 34.2 Mb) with variants in a 5-Mb window on chromosome (chr) 20 (32.5–37.5 Mb; GRCh38 coordinates) in CEU and GBR 1KGP populations.

To identify structural variation at *ASIP* that could underlie genetic associations with pigmentation, we examined long-read sequence assemblies generated by the Human Genome Structural Variation Consortium^23^ (HGSVC2; *n* = 32). The single individual that was heterozygous for the light-pigmentation/cancer-risk haplotype (NA12329) was also heterozygous for a large, 3.3-kb structural variant in an intron of *ASIP* (Fig. 1a,b) previously inferred from short-read sequencing analyses^24,25^. Optical mapping data (Bionano) confirmed this insertion as the only large structural variant within 500 kb of *ASIP* carried by NA12329. The variant overlaps a SINE-VNTR-Alu (SVA) element annotated by RepeatMasker^26^ (in antisense orientation relative to *ASIP* transcription) at chr20:34228123–34231419 in the GRCh38 reference, suggesting that the human genome (GRCh38) reference sequence has an allele with a recent, polymorphic SVA insertion that is in fact absent from most *ASIP* haplotypes. SVA elements are an active, recent family of retrotransposons unique to great apes, with the E, F and F_1_ subfamilies specific to humans^27^. Sequence evidence suggests that this SVA is in the youngest SVA F_1_ subfamily^28,29^, as it lacks a key 5′ (CCCTCT)n hexameric repeat and Alu-like elements and also contains 92 bp matching the *MAST2* exon 1 that was 5′-transduced into the subfamily’s source element. Notably, this polymorphic SVA is 3.9 kb downstream of (and in the opposite orientation to) another, nonpolymorphic 1.6-kb SVA F retrotransposon within the same intron of *ASIP* (Fig. 1b).

To facilitate deeper analysis of this variant in larger, phenotyped cohorts, we devised a strategy for ascertaining individual-level genetic states (genotypes) from short-read whole-genome sequencing (WGS) alignment patterns specific to each allele (Fig. 1c; Methods). Applying this approach to high-coverage WGS of 1000 Genomes Project (1KGP) samples^30^ demonstrated good separation of genotype clusters (Fig. 1d). Across 1KGP population samples, the SVA F_1_ insertion exhibited the highest allele frequencies (7–8%) in the northwest European (GBR and CEU) population samples and was not detected in (nonadmixed) samples of a variety of populations from Africa and Asia (Fig. 1e). In CEU and GBR population samples, the SVA F_1_ insertion was in strong linkage disequilibrium (LD) (*r*^2^ = 0.93) with the pigmentation-associated index single nucleotide polymorphism (SNP) rs6059655, and LD with other SNPs spanned a ~5-Mb extended haplotype (Fig. 1f).

**Fig. 2:**
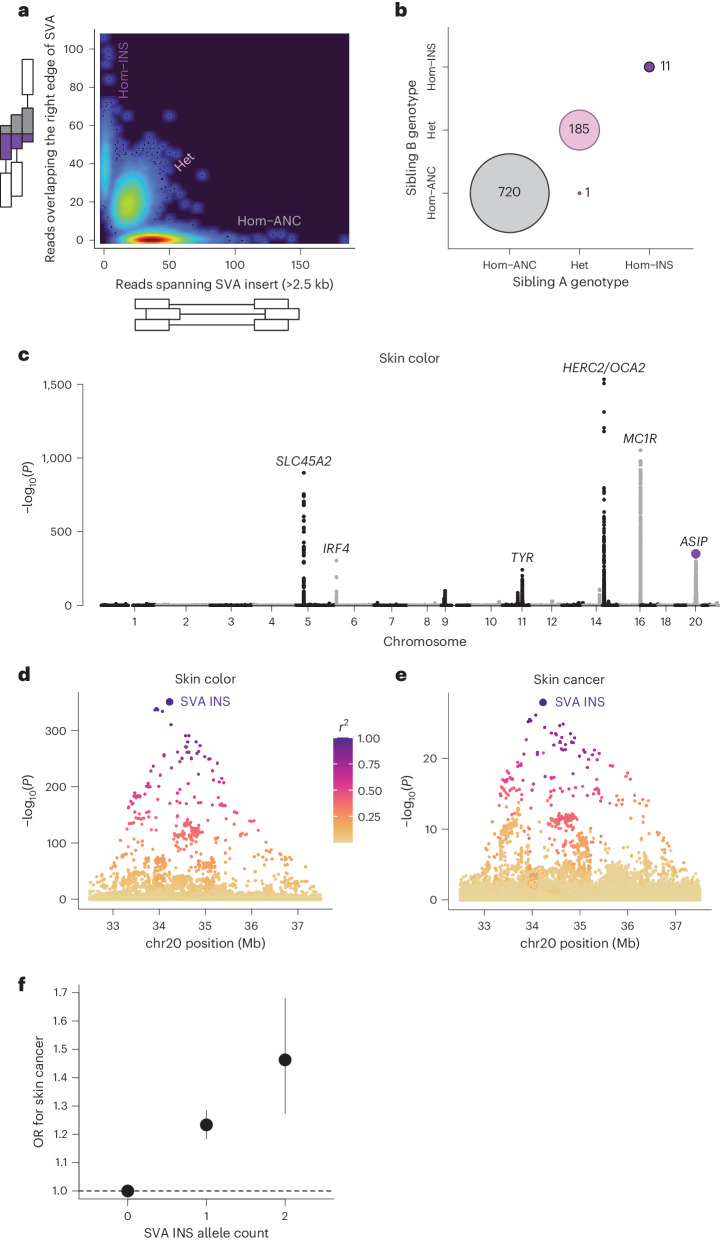
Association of SVA F_1_ insertion with pigmentation phenotypes in UKB. **a**, Genotyping of 199,956 UKB participants with WGS. Three genotype clusters corresponding to the dosage of the SVA F_1_ insertion are apparent. **b**, Genotype concordance of 917 sibling pairs sharing both *ASIP* haplotypes IBD2. All but one of the sibling pairs agree on the genotype call made for the SVA F_1_ insertion. **c**, Associations of genome-wide imputed SNP and indel variants with self-reported skin color, coded on a scale from fairest to darkest (*n* = 167,568); *P* values are from linear regression. The association of the SVA F_1_ insertion is plotted in purple. **d**, Local association plot for skin color (*n* = 167,568) at the extended *ASIP* locus. Association strengths track with LD with the SVA F_1_ insertion (yellow-to-purple shading), indicated by the large purple dot. **e**, Associations at *ASIP* with any type of skin cancer (C43 or C44 ICD-10 code; *n*_controls_ = 154,340 and *n*_cases_ = 15,295). **f**, ORs for skin cancer risk for individuals heterozygous (*n* = 31,932) or homozygous (*n* = 1,952) for the SVA F_1_ insertion relative to individuals homozygous for no insertion (*n* = 135,751). Centers, effect size estimate for each genotype from logistic regression; error bars, 95% CIs.

**Extended Data Fig. 1:**
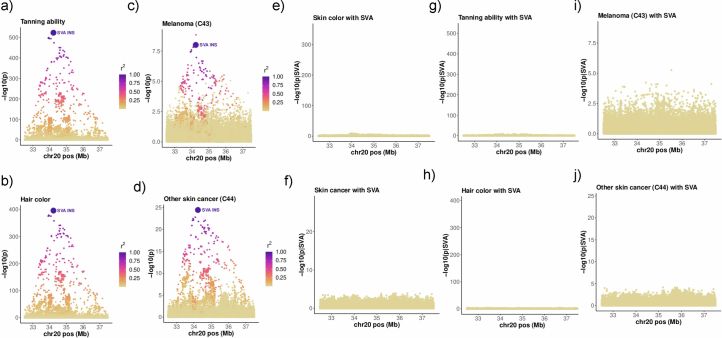
Associations of SVA F_1_ insertion and nearby variants to pigmentation phenotypes in UK Biobank. **a-d**, Local association plots in a 5-Mb window surrounding *ASIP* for (**a**) self-reported tanning ability (*n* = 166,404), (**b**) self-reported hair color (*n* = 167,310), (**c**) melanoma (C43 ICD-10 code, *n* = 169,635), and (**d**) other skin cancers including basal and squamous cell carcinomas (C44 ICD-10 code, *n* = 169,635). Association strengths track with linkage disequilibrium with the SVA F_1_ insertion (yellow-to-purple shading), indicated by the large purple dot. **e-j**, Conditional association plots for SNPs and indels after including SVA F_1_ genotype as a covariate for (**e**) self-reported skin color (*n* = 167,568), (**f**) any skin cancer (C43 or C44 ICD-10 codes, *n* = 169,635), (**g**) self-reported tanning ability (*n* = 166,404), (**h**) self-reported hair color (*n* = 167,310), (**i**) melanoma (C43 ICD-10 code, *n* = 169,635), and (**j**) other skin cancers including basal and squamous cell carcinomas (C44 ICD-10 code, n = 169,635).

**Extended Data Fig. 2:**
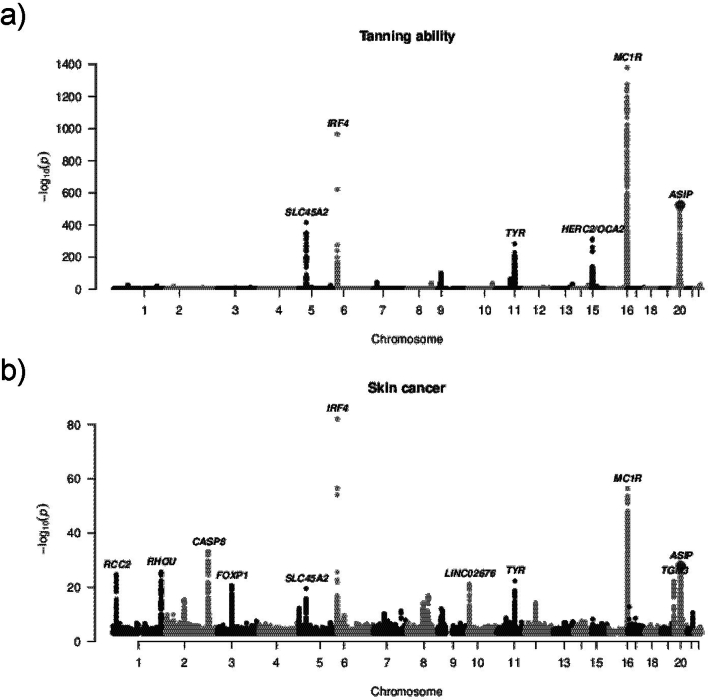
Genome-wide associations with tanning ability and skin cancer risk in UK Biobank. **a**, Associations from linear regression across all imputed variants with self-reported tanning ability (*n* = 166,404). **b**, Associations from linear regression across all imputed variants with any type of skin cancer (union of C43 and C44 ICD-10 codes, *n* = 169,635).

We applied the same SVA genotyping approach to WGS data available for 169,641 unrelated White individuals of European genetic ancestry in the deeply phenotyped UK Biobank (UKB) cohort^31,32^ (Fig. 2a), finding the allele frequency of the insertion to be 11%. We estimated the accuracy of genotyping to be *r*^2^ ≈ 0.997 based on the concordance of genotype calls across sibling pairs sharing both *ASIP* alleles identically by descent (IBD2) (Fig. 2b). Across pigmentation traits including skin color, hair color and tanning response, the SVA F_1_ insertion associated more strongly with lighter pigmentation (0.22 [95% confidence interval (CI):0.21–0.23], 0.24 [0.23–0.25] and 0.27 [0.26–0.28] s.d.; *P* = 2.8 × 10^−351^, 2.0 × 10^−396^ and 1.5 × 10^−523^, respectively) than did all other SNP and indel variants in the region, explaining 0.9–1.4% of trait variance (Fig. 2c,d and Extended Data Figs. 1a,b and 2a). Likewise, the SVA F_1_ insertion associated more strongly with increased skin cancer risk (odds ratio (OR) = 1.23 [1.18–1.27]; *P* = 1.3 × 10^−28^) than did any nearby variant (Fig. 2e,f and Extended Data Figs. 1c,d and 2b). In a joint analysis (of both the SVA F_1_ insertion and the lead SNP rs6059655) for tanning response (the pigmentation trait with the strongest association at the locus), the SVA F_1_ insertion remained significantly associated (*P* = 6.9 × 10^−31^), whereas the lead SNP did not (*P* = 0.56). Fine-mapping analysis using SuSiE^33^ also selected the SVA as the only member of a single credible set. Conditional association analyses including the SVA as a covariate further suggested that the SVA F_1_ insertion almost completely accounted for pigmentation associations at the locus: residual signal was only 1–2% as strong (Extended Data Fig. 1e–j).

**Fig. 3:**
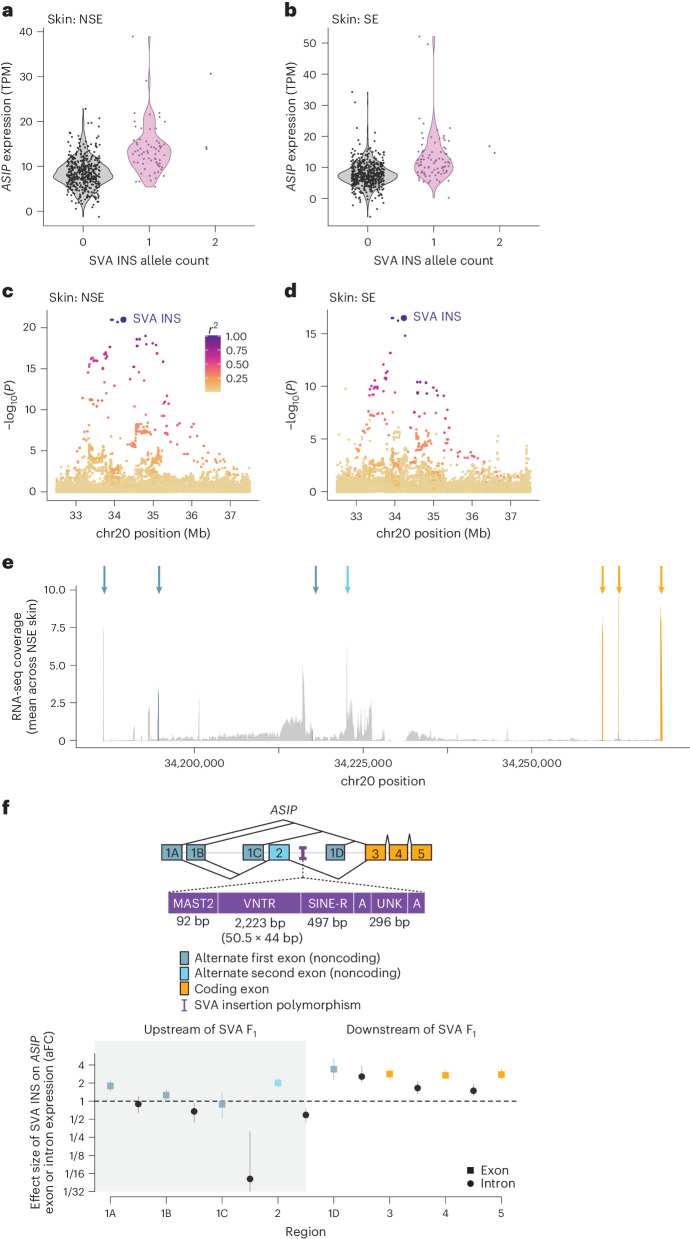
Association of SVA F_1_ insertion with expression of *ASIP* exons and introns in skin. **a**, *ASIP* gene expression in GTEx NSE skin samples (*n* = 517) stratified by SVA F_1_ insertion genotype. **b**, Analogous to **a**, for GTEx SE skin samples (*n* = 605). **c**, Local association plot for *ASIP* gene expression in NSE skin samples (*n* = 517). Association strengths track with LD with the SVA F_1_ insertion (yellow-to-purple shading). **d**, Analogous to **c**, for GTEx SE skin samples (*n* = 605). **e**, Depth of coverage of RNA-seq read alignments at *ASIP*, averaged across NSE skin samples. Coverage at exons is indicated with colors corresponding to the *ASIP* gene model below (dark blue, alternative first 5′ UTR exons; light blue, optional second 5′ UTR exon; gold, coding exons). RNA-seq coverage in introns is indicated in gray. **f**, Effect size of SVA F_1_ insertion for expression of each *ASIP* exon and intron (in units of allelic fold change; aFC) in NSE skin samples (*n* = 517). Intronic regions are defined between adjacent exons; measurements in these regions presumably correspond to prespliced mRNA, potentially from several isoforms. Centers, point estimate of aFC from linear regression coefficients for the indicated region; error bars, 95% CIs from bias-corrected and accelerated bootstrap.

**Extended Data Fig. 3:**
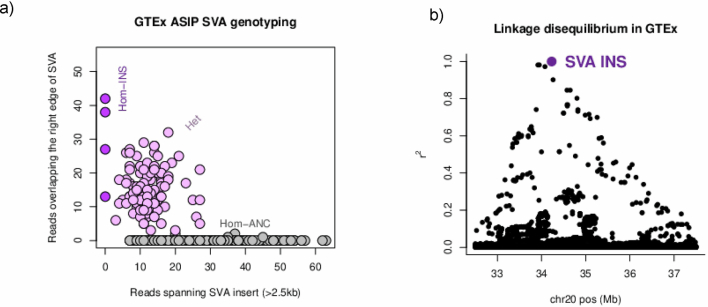
Genotyping of SVA F_1_ insertion in GTEx cohort. **a**, Genotyping of 838 GTEx donors with whole-genome sequencing. **b**, Linkage disequilibrium (*r*^2^) of the SVA F_1_ insertion with variants between 32.5 Mb to 37.5 Mb on chromosome 20 across GTEx donors.

**Extended Data Fig. 4:**
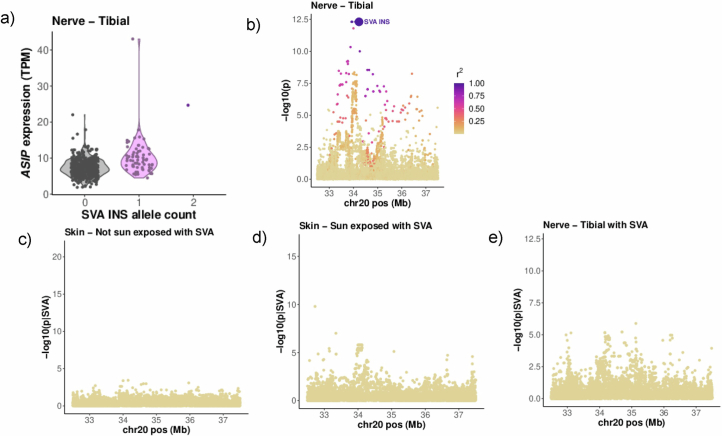
Associations of SVA F_1_ insertion and nearby variants to expression of *ASIP* in skin and tibial nerve. **a**, *ASIP* gene expression in GTEx tibial nerve samples (*n* = 532), stratified by SVA F_1_ insertion genotype. Tibial nerve was the only other tissue that appeared to have evidence of the same eQTL. TPM, transcripts per million. **b**, Local association plot for *ASIP* gene expression in tibial nerve samples (*n* = 532) in the region 32.5 Mb to 37.5 Mb on chromosome 20. Association strengths track with linkage disequilibrium with the SVA F_1_ insertion (yellow-to-purple shading), indicated by the large purple dot. **c**, Conditional association plot for *ASIP* gene expression in GTEx skin (not sun-exposed, NSE) samples (*n* = 517) after including SVA F_1_ genotype as a covariate. **d**, As in **c**, but for skin (sun-exposed, SE) samples (*n* = 605). **e**, As in **c**, but for tibial nerve samples (*n* = 532).

As SNPs on this haplotype have been observed to associate with *ASIP* expression levels in skin^11^, we next asked whether this insertion is the likely cause of this effect on *ASIP* expression. Genotyping the SVA F_1_ insertion in WGS data available for tissue donors of the Genotype-Tissue Expression (GTEx) Project^34^ (Extended Data Fig. 3) showed that, as with the pigmentation associations, the insertion associated strongly with *ASIP* expression in both sun-exposed (SE) and not sun-exposed (NSE) skin (*P* = 3.5 × 10^−17^ and 1.3 × 10^−21^, respectively), and that the insertion appeared to account for most of the eQTL signal at the locus (Fig. 3a–d and Extended Data Fig. 4). The SVA insertion associated with a 2.2-fold (1.9–2.6) increase in *ASIP* expression in NSE skin. Closer examination of RNA sequencing (RNA-seq) read alignments at *ASIP* showed substantial RNA-seq coverage at several alternative first exons as well as within introns (Fig. 3e). Whereas the SVA F_1_ insertion associated with broadly increased expression across all exons, it associated with decreased abundance of unspliced transcripts containing intronic sequence upstream of the SVA (Fig. 3f).

**Fig. 4:**
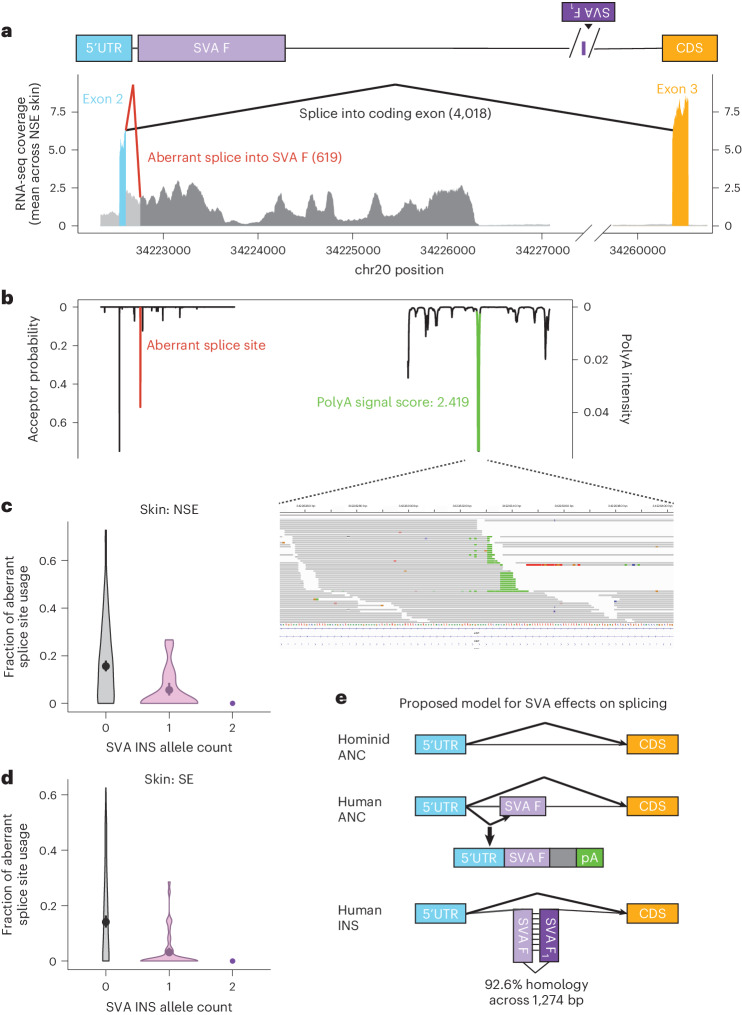
Aberrant splicing and early polyadenylation of *ASIP* transcripts from haplotypes without SVA F_1_ insertion. **a**, RNA-seq coverage depth at *ASIP* (averaged across NSE skin samples), annotated with splice junctions from the exon 2 (5′ UTR) splice donor. Most split reads support the canonical splice junction to exon 3 (*n* = 4,018 reads; black junction), but a substantial minority support an aberrant splice junction into an acceptor site in a nearby (nonpolymorphic) SVA F element (*n* = 619 reads; red junction). **b**, Computationally predicted splice acceptors (SpliceAI^36^) and polyadenylation signals (APARENT^37^). Inset, RNA-seq read alignments containing soft-clipped poly(A) sequences at the predicted polyadenylation peak. **c**, Fraction of splice junctions from exon 2 that aberrantly splice into the acceptor site in the nearby SVA F element (versus splicing to exon 3), stratified by SVA F_1_ insertion genotype in GTEx NSE skin samples. Only samples with greater than ten total reads supporting either splice junction are included in the violin plots (*n* = 131) to reduce noise from less informative samples. Centers, combined fraction of aberrant splicing across all samples with each SVA F_1_ insertion genotype (total *n* = 517); error bars, 95% CIs from bias-corrected and accelerated bootstrap. **d**, Analogous to **c**, for GTEx SE skin samples (*n* = 158 for violin plots). Centers, combined fraction of aberrant splicing across all samples with each SVA F_1_ insertion genotype (total *n* = 605); error bars, 95% CIs from bias-corrected and accelerated bootstrap. **e**, Proposed model for the effects of the ancient SVA F insertion and the recent SVA F_1_ insertion on splicing patterns of *ASIP* transcripts. The original hominid ancestral allele—lacking either retrotransposon—splices normally between alternate noncoding exon 2 and coding exon 3. Insertion of the SVA F element then causes a fraction of *ASIP* transcripts to splice aberrantly to the introduced acceptor site, leading to early polyadenylation. The subsequent SVA F_1_ insertion then restores normal splicing to coding exon 3 in all transcripts, possibly by sequestering the splice acceptor or splice enhancer motifs.

**Extended Data Fig. 5:**
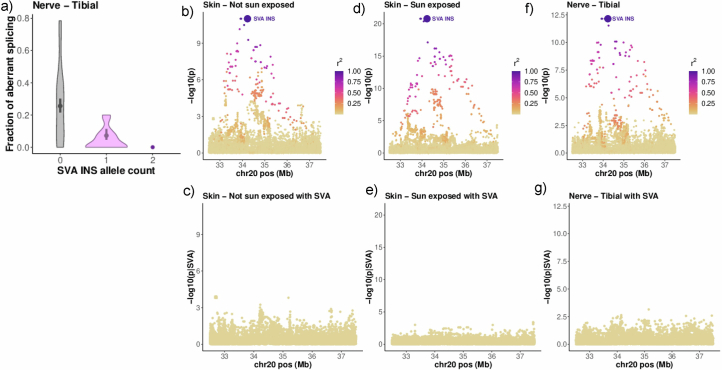
Associations of SVA F_1_ insertion and nearby variants to aberrant *ASIP* splice junction usage in skin and tibial nerve. **a**, Fraction of splice junctions from exon 2 that aberrantly splice into the acceptor site in the nearby SVA F element (versus splicing to exon 3), stratified by SVA F_1_ insertion genotype in GTEx tibial nerve samples. Only samples with greater than 10 total reads supporting either splice junction are included in the violin plot (*n* = 33) to reduce noise from less informative points. Centers: combined fraction of aberrant splicing across all samples with each SVA F_1_ insertion genotype (total *n* = 532); error bars: 95% CIs from bias-corrected and accelerated bootstrap. **b**, Local association plot for aberrant splice junction usage in skin (not sun-exposed, NSE) samples (*n* = 433) in the region 32.5 Mb to 37.5 Mb on chromosome 20. Association strengths track with linkage disequilibrium with the SVA F_1_ insertion (yellow-to-purple shading), indicated by the large purple dot. **c**, As in **b**, but after including SVA F_1_ genotype as a covariate. **d**, As in **b**, but for skin (sun-exposed, SE) samples (*n* = 497). **e**, As in **d**, but after including SVA F_1_ genotype as a covariate. **f**, As in **b**, but for tibial nerve samples (*n* = 364). **g**, As in **f**, but after including SVA F_1_ genotype as a covariate.

We therefore hypothesized that the SVA F_1_ insertion increases *ASIP* expression by improving splicing fidelity (and thus reducing the ascertainment of unspliced transcripts). To test this idea, we analyzed all the *ASIP* splice junctions observed in GTEx skin samples (reported by LeafCutter^35^). One of the more frequent anomalous splice events involved splicing from an *ASIP* 5′ untranslated region (UTR) exon to a computationally predicted splice acceptor (SpliceAI^36^ acceptor probability of 0.51) within the nonpolymorphic SVA F element that resides in the same intron (in opposite orientation) as the polymorphic SVA F_1_ insertion (Fig. 4a,b). In heterozygotes for the SVA F_1_ insertion, the relative frequency of splicing into the SVA F element, rather than the downstream coding exon, decreased from 14.1% to 3.1% in SE skin and 15.6% to 5.6% in NSE skin (*P* = 1.6 × 10^−21^ and 1.0 × 10^−11^, respectively; Fig. 4c,d). In three GTEx donors homozygous for the SVA F_1_ insertion, no evidence of splicing into the SVA F element was observed. As with the expression QTL, the SVA F_1_ insertion seemed to explain this splicing QTL signal (Extended Data Fig. 5). Scanning downstream to determine the fate of these aberrant transcripts revealed a termination point of the intronic read alignments, at which several reads ended in poly(A) sequences (Fig. 4a,b), concordant with a polyadenylation site predicted by APARENT^37^ (Fig. 4b). These observations together indicate that the aberrant transcripts spliced into the upstream SVA F element are terminated to yield a noncoding transcript, and that the presence of the polymorphic SVA F_1_ insertion inhibits the production of such transcripts while increasing the production of ASIP-coding transcripts. Because translation stabilizes transcripts, this analysis may underestimate the relative amount of noncoding transcript being produced, as suggested by the fold increase in expression. We propose that the polymorphic SVA F_1_ element—inserted in inverse orientation relative to the upstream SVA F element—forms (together with the upstream SVA F) a hairpin structure that blocks the function of splice enhancers and/or splice acceptor sequences in the SVA F element, ensuring productive splicing to ASIP-coding exons downstream of the SVA insertions (Fig. 4e). This model resembles recent reports of inverted pairs of Alu elements modulating splicing via formation of an RNA hairpin^3,38^.

**Extended Data Fig. 6:**
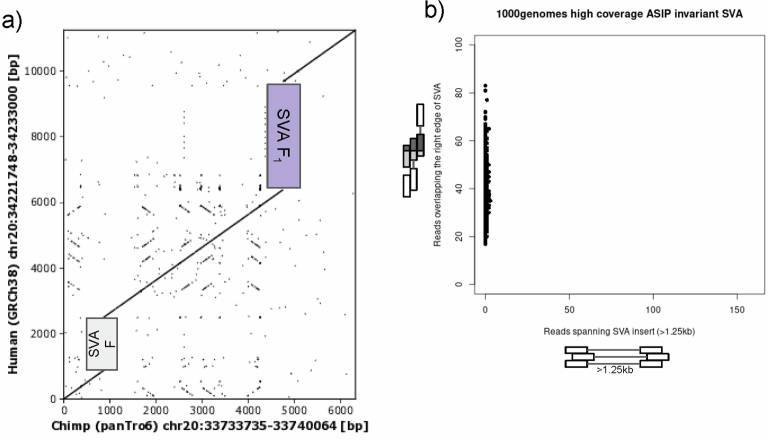
Characterization of non-polymorphic SVA F element upstream of SVA F_1_ insertion. **a**, Pairwise sequence alignment dot plot of GRCh38 human reference vs. panTro6 chimpanzee reference at *ASIP*. The human reference contains an SVA F element upstream of the polymorphic SVA F_1_ insertion, neither of which are present in chimpanzee. **b**, Assessment of SVA F presence in 1KGP individuals. Similar to the genotyping approach we used for the polymorphic SVA F_1_ insertion, we counted reads overlapping the right edge of the SVA F element (indicating presence of at least one allele containing the SVA F) and discordant reads with a fragment size approaching the length of the SVA F element (1.6 kb) (which would indicate the presence of an allele lacking the SVA F). All individuals in 1KGP appear to carry the SVA F on both *ASIP* alleles.

**Extended Data Fig. 7:**
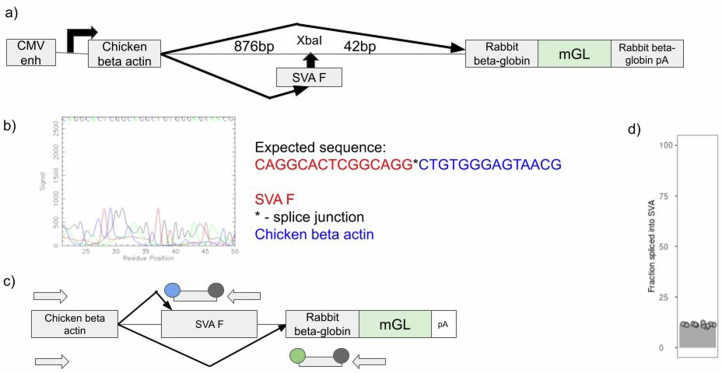
Construct to measure splicing into upstream SVA F element *in vitro.* **a**, Design of base construct, pCAG-mGL, and relative position of introduced SVA F sequence in the hybrid intron of the CAG promoter at XbaI restriction site. **b**, Sanger sequencing results from transcripts produced by pCAG-mGL_SVA construct and match to the expected sequence that would arise from splicing from the upstream chicken beta-actin exon (in blue) to the aberrant splice acceptor within the SVA F element (in red) observed in GTEx RNA-seq at *ASIP*. Note that the sequence is antisense to the transcript. **c**, Design of RT-ddPCR assays to measure relative splicing from the chicken beta-actin exon to introduced splice acceptor in SVA F versus downstream rabbit beta-globin exon. The arrows represent forward and reverse primers and the rectangles with circles represent quenched fluorescently labeled probes, where the blue circle is a FAM fluorophore, the green circle is a HEX fluorophore, and the gray circles are quenchers that are cleaved during polymerase extension. **d**, Fraction of splicing into the introduced SVA F element in pCAG-mGL_SVA construct (*n* = 12 replicates). Each point indicates the measured value in a replicate, with the bar indicating the mean fraction.

These results led us to hypothesize that ancient *ASIP* alleles that predated both SVA insertions spliced *ASIP* more similarly to the splicing yielded by the present-day derived haplotype that contains both SVAs. Human genetic data do not enable assessment of this question because the upstream SVA F element, which is human-specific (Extended Data Fig. 6a), has reached fixation in present-day human populations (Extended Data Fig. 6b). We therefore used an in vitro construct to verify that the SVA F element can function as a splicing acceptor when inserted in the hybrid intron of the CAG promoter^39^ (Extended Data Fig. 7a; Methods), similar to SVA splicing constructs from previous work^26^. Insertion of the SVA F element caused approximately 11% of transcripts to splice into the SVA at the same aberrant acceptor site as in *ASIP* (Extended Data Fig. 7b–d), consistent with the idea that the ancient human (no longer polymorphic) SVA insertion had reduced productive splicing of *ASIP*.

**Fig. 5:**
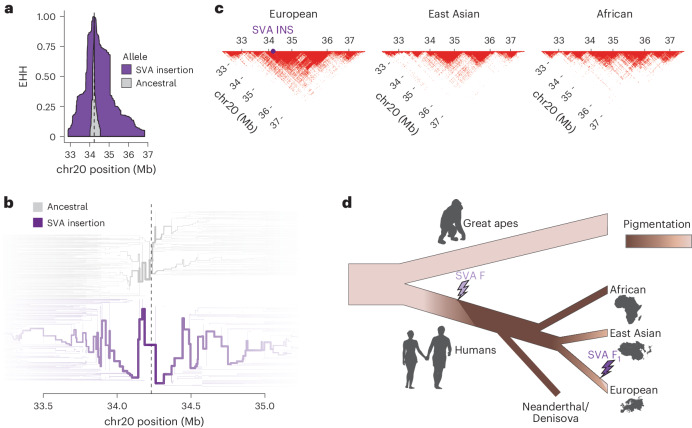
Recent selection for the SVA F_1_ insertion haplotype in ancestral European populations. **a**, Extended haplotype homozygosity (EHH) plot for haplotypes with and without the SVA F_1_ insertion in UKB (*n* = 169,641). The EHH value at a given variant is the probability that two haplotypes are homozygous at all variants between it and the focal variant^49^ (here, the SVA F_1_ insertion). Haplotypes with the SVA F_1_ insertion have higher EHH in both directions, suggesting recent positive selection on the allele. **b**, Haplotype bifurcation diagram depicting haplotypes with and without the SVA F_1_ insertion (1,000 haplotypes per group, selected randomly from the UKB analysis set). Bifurcations indicate SNPs that distinguish haplotypes, and line weights indicate proportions of haplotypes that carry each SNP allele. This diagram provides a haplotype-level representation of the comparatively reduced number of recombination events that have occurred on haplotypes containing the SVA F_1_ insertion. **c**, LD in 5-Mb region surrounding *ASIP* (32.5–37.5 Mb; GRCh38 coordinates) in 1KGP superpopulations (excluding admixed African populations). For each superpopulation, 13,000 SNP/indel variants with MAF > 1% were sampled, and the LD plot displays a red point for each pair of variants with *r*^2^ > 0.2. Haplotypes sampled in populations with European genetic ancestry (*n* = 1,006) exhibit excess LD between variants in this region compared with African (*n* = 1,002) or East Asian (*n* = 1,008) haplotypes. The purple point indicates the relative position of the SVA F_1_ insertion in European haplotypes. **d**, Evolution of hair and skin pigmentation in hominid lineages, with relative timing and pigmentation effects of each SVA insertion highlighted. Ancestral hominids and many extant great apes have light skin pigmentation, with UV protection conferred by denser body hair. Early human evolution involved increasing pigmentation and decreasing body hair. The ancient SVA F retrotransposon, which is shared by Neanderthals with modern humans on all continents, may have inserted during this period into the *ASIP* intron—decreasing *ASIP* expression and increasing pigmentation—and became fixed in *Homo sapiens*. Much more recently, a subsequent SVA F_1_ insertion appeared and expanded in frequency (to several percent) within ancestral European populations, increasing *ASIP* expression and decreasing pigmentation.

**Extended Data Fig. 8:**
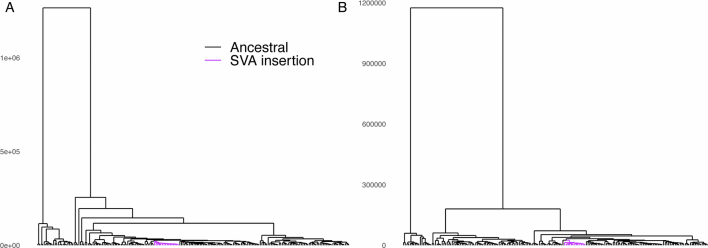
Genealogy of haplotypes at the *ASIP* SVA F_1_ insertion. **a**,**b**, Coalescent trees estimated by Relate for (**a**) CEU (*n* = 202) and (**b**) GBR (*n* = 186) haplotypes at the SVA F_1_ insertion site. The purple branch contains all haplotypes carrying the SVA F_1_ insertion. Age (in years) on the *y*-axis assumes a generation time of 28 years.

In contrast to the nonpolymorphic SVA F element, the polymorphic SVA F_1_ insertion allele (the reference, minor allele) was detected only in population samples with European genetic ancestry. Furthermore, this ASIP-expression-increasing, pigmentation-decreasing SVA F_1_ insertion allele exhibited long-range (>3 Mb) LD—generally a property of recent mutations—on European haplotypes (Fig. 1f and Fig. 5a,b), whereas such long-range LD was not observed in non-European population samples (Fig. 5c). Analysis of haplotype genealogies in 1KGP CEU and GBR population samples (Methods) dated the insertion at 16,400–21,800 years ago and 14,300–25,400 years ago, respectively (Extended Data Fig. 8). These lines of evidence suggest that this SVA insertion has increased quickly in allele frequency relative to other European haplotypes at the *ASIP* locus, reaching up to 11% allele frequency in some European populations in a short period of time.

The timing and effects of these two retrotransposon insertions at *ASIP* seem broadly consistent with early and recent changes in human pigmentation. The ancient SVA F insertion—which, despite being relatively young within the SVA F family (Methods), is present in Neanderthal genomes^40–43^ (Supplementary Figs. 1 and 2)—probably contributed to increases in pigmentation early in human evolution, potentially helping to enable humans’ concomitant loss of body hair. As modern humans later migrated around the world, pigmentation-lightening alleles appear to have emerged in several settings with reduced exposure to ultraviolet light^44,45^. The much more recent SVA F_1_ insertion seems to have contributed to decreased pigmentation in a subset of individuals within ancestral European populations, while also leading to an increase in sunburn frequency (OR = 1.34 [1.30–1.37]; *P* = 1.4 × 10^−105^) and skin cancer risk (Fig. 5d).

*ASIP* thus appears to provide an example of how a sequence of retrotransposon insertions at a single locus can modulate phenotype several times in a species’ recent history. The fact that the effect of such a common, large polymorphism (3.3 kb) could remain unnoticed for 15 years (even after recent advances in retrotransposon association analysis^2^) speaks to the importance of fully integrating structural variants into genetic association analyses. Interestingly, on evolutionary timescales, expression of *ASIP* and its homologs seems to have been modulated primarily by structural mutations that caused pigmentation changes in diverse species^18–22^. It is interesting to consider the possibility that some loci could be prone, across tens of millions of years, to evolve via structural variation.

**Extended Data Fig. 9:**
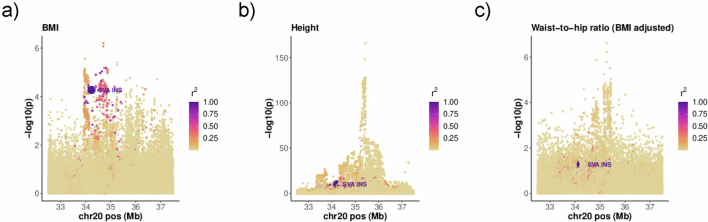
Associations of SVA F_1_ insertion and nearby variants to anthropometric phenotypes in UK Biobank. **a**-**c**, Local association plots in a 5-Mb window surrounding *ASIP* for (**a**) BMI (*n* = 169,052), (**b**) height (*n* = 169,239), and (**c**) waist-hip ratio adjusted for BMI (*n* = 169,285). Only the associations with height reach genome-wide significance, but the association pattern does not appear to colocalize with linkage disequilibrium with the SVA F_1_ insertion (yellow-to-purple shading).

The causal mechanism we have proposed—in which the recent SVA F_1_ insertion acts by mitigating a splicing hypofunction introduced by an ancient SVA F insertion—should be testable by future experiments that insert the SVA F_1_ element into cell types that express *ASIP*. Increasingly available single-cell RNA-seq data may help identify the responsible cell type, which previous work has suggested could be fibroblasts or melanocyte precursors in the dermis^11,46^. While a recent study found that ectopic expression of *ASIP* (due to a rare *ITCH–ASIP* gene fusion) yields a monogenic phenotype including obesity, overgrowth and light pigmentation^47^, the common SVA insertion polymorphism had little-to-no association with anthropometric traits in UKB (Extended Data Fig. 9) despite ample power to find associations, and its effect on *ASIP* expression (in GTEx) was observed only in skin and tibial nerve tissues (Fig. 3 and Extended Data Figs. 4 and 5) (possibly reflecting shared descent of melanocytes and Schwann cells from a common neural crest-derived progenitor^48^). More broadly, these results highlight the potential for integrative analyses of newly available genetic data resources to yield new insights, even in loci that have been well studied.

## Methods

### HGSVC2 genetic data

PacBio long-read assembly contigs and Bionano structural variant calls for individuals in HGSVC2 were downloaded from the 1KGP FTP site (ftp://ftp.1000genomes.ebi.ac.uk/vol1/ftp/data_collections/HGSVC2/release/v1.0/assemblies/ and ftp://ftp.1000genomes.ebi.ac.uk/vol1/ftp/data_collections/HGSVC2/working/20200219_Bionano_optical_map_SVs/). Assembled sequences around *ASIP* were extracted by building each set of haploid contigs (h1 and h2) into a BLAST database and using a region from *ASIP* lacking repetitive sequences (chr20:34224248–34225005 in GRCh38) as a search query for BLASTN (v.2.12.0). Dotplots were generated with FlexiDot (v.1.06)^50^.

### 1KGP genetic data

High-coverage data for 2,504 unrelated 1KGP individuals^30^ was sliced to obtain paired reads mapping to the genomic interval chr20:34221626–34232418. To genotype the SVA F_1_ element present in the reference genome at chr20:34228123–34231419, we counted (1) discordant read pairs aligned within a window extending 1 kb in each direction from the SVA F_1_ with template length (TLEN) exceeding 2.5 kb (indicative of the presence of the major allele not containing the SVA F_1_); and (2) reads spanning the right breakpoint (chr20:34231418) (indicative of the minor allele with the SVA F_1_). Individuals were genotyped as homozygous for having the SVA F_1_ insertion (Hom-INS) if the number of reads overlapping the right breakpoint (*n*_INS_) was greater than three times the number of reads with TLEN >2.5 kb (*n*_ANC_), that is, *n*_INS_ > 3*n*_ANC_. Individuals were genotyped as homozygous for not having the insertion (Hom-ANC) if the number of reads overlapping the right breakpoint was <0.25× the number of reads with TLEN >2.5 kb, that is, *n*_INS_ < 0.25*n*_ANC_. The remaining individuals were genotyped as heterozygous for the insertion. This strategy was necessitated by low mappability throughout the SVA F_1_ element, which precluded read-depth analysis in the region. Similarly, to search for individuals that could in theory lack the upstream SVA F element at chr20:34222626–34224238, we counted (1) discordant read pairs aligned within a window extending 1 kb in each direction from the SVA F with TLEN exceeding 1.25 kb (which would suggest the presence of an allele not containing the SVA F); and (2) reads spanning the right breakpoint (chr20:34224238) (indicative of the expected SVA F sequence).

Pairwise LD plots (Fig. 5c) were generated for each of the European, East Asian and African genetic ancestry superpopulations by first extracting variants in the region chr20:32.5–37.5 Mb (GRCh38 coordinates) with population minor allele frequency (MAF) >1% with bcftools (v.1.14)^48^. ASW and ACB populations were excluded from the African genetic ancestry set to avoid selecting variants that would have excessively long linkage due to recent admixture. For each superpopulation, 13,000 variants were then sampled randomly to yield even density in plotting, and pairwise *r*^2^ matrices were computed with plink1.9 (v.1.90b6.26)^51^.

### UKB genetic and phenotype data

Genotyping of the SVA F_1_ insertion polymorphism was performed on read alignments at *ASIP* extracted from WGS data available for 199,956 UKB participants^32^. The same overall genotyping approach as above was used for UKB, with the slight modification that individuals were genotyped as Hom-INS if the read categories from before satisfied *n*_INS_ > 10 + 3*n*_ANC_ and as Hom-ANC if *n*_INS_ < 2 + 0.2*n*_ANC_. Different linear separators were used here based on observed differences in the relative presence of *n*_INS_ and *n*_ANC_ for each genotype, presumably due to slight differences in sequencing and alignment parameters (for example, average coverage, fragment length, bwa-mem options). In this much larger sample, a third genotype cluster with few discordant read pairs and many reads overlapping the right breakpoint (indicating homozygosity for the SVA F_1_ insertion allele) became clearly defined. Because the UKB data set contains several hundred sibling pairs that share both haplotypes IBD2 in this region (based on at most three mismatching SNP-array genotypes within a 2-Mb window centered at *ASIP*, computed using plink1.9 –genome), we could correlate genotype calls made between these sibling pairs to estimate genotyping accuracy.

From these 199,956 individuals, we first removed 11,953 who did not report White ethnicity. A further 18,027 individuals were then filtered to remove outliers (>6 s.d.) across the first ten genetic ancestry principal components (PCs) and to select one individual from each first- or second-degree related pair, as described^52^. An additional 335 individuals were then excluded who had WGS but were not present in the imp_v3 imputed genotype dataset^31^. This set of 169,641 individuals was then used for both genome-wide association analyses with BOLT-LMM (v.2.4.1)^53^ as well as local association analyses with plink2 (v.2.00a3.7; Intel AVX2)^51^ (see below). Phenotypes of self-reported pigmentation traits (tanning ability, skin color, hair color, childhood sunburn frequency) were obtained from UKB touchscreen questionnaire datafields, adjusting the coding of hair color to assign red hair a value of 1 and blonde hair a value of 2 to better follow the order corresponding to increasing eumelanin:pheomelanin ratio^54^ and binarizing the sunburn phenotype to individuals with no instances of severe childhood sunburns and those with at least one instance. Phenotypes of anthropometric traits (height, body mass index (BMI), waist-hip ratio adjusted for BMI) were processed as previously described^55^. Phenotypes of skin cancer diagnoses (derived from cancer registry data, accessed 26 October 2022) were taken from the 10th revision of the International Statistical Classification of Diseases and Related Health Problems (ICD-10) codes C43 (melanoma), C44 (nonmelanoma) and the union of the two for all skin cancers (C43 + C44).

### UKB: local association analyses at *ASIP*

VCF files containing genotype calls from UKB WGS were processed by first splitting multiallelic variants into separate biallelic variants with bcftools. Association analyses were performed on variants with MAF > 0.001 using plink2 (v.2.00a3.7; Intel AVX2) using linear regression for quantitative traits (tanning ability, skin color, hair color, height, BMI and waist-hip ratio adjusted for BMI) and logistic regression for binary skin cancer traits (C43, C44 and C43 + C44) with standard covariates (age, age squared, sex, 20 genetic PCs and assessment center). LD with the SVA F_1_ (*r*^2^) was computed with plink1.9 (v.1.90b6.26).

### UKB: genome-wide association analyses

Genome-wide association analyses of skin color, tanning ability and any skin cancer (C43 + C44) were performed on imputed variants (imp_v3) for the same 169,641 individuals using linear regression with BOLT-LMM (v.2.4.1). The above covariates as well as genotyping array were included as covariates, and variants were filtered to MAF > 0.001 and INFO > 0.3. Manhattan plots were generated using variants with *P* < 0.01 using the qqman (v.0.1.8) package^56^.

### UKB: fine-mapping of phenotype associations

The 5,000 top associating variants from the region chr20:32.5–37.5 Mb (GRCh38) were extracted from VCF files containing genotype calls from UKB WGS (again splitting multiallelic variants into separate biallelic variants with bcftools). Standard covariates (age, age squared, sex, 20 genetic PCs and assessment center) were regressed out from both phenotype and genotypes and provided as input to the susieR (v.0.12.35) package^33^ for fine-mapping (allowing up to *L* = 10 causal variables).

### UKB: measuring extended haplotype homozygosity

SVA F_1_ diploid genotypes were phased onto a prephased scaffold of SNP-array genotyped variants for the same 169,641 individuals using the phase_common tool from SHAPEIT5 (v.5.1.1)^57^. The SNP-array genotyped variants were then matched with variants present in the low-coverage 1KGP3 (ref. ^58^) VCF, for which ancestral and derived alleles had been annotated previously. These phased haplotypes were then used to measure extended haplotype homozygosity (EHH) and generate bifurcation plots comparing haplotypes with and without the SVA F_1_ insertion with the rehh (v.3.2.2) package^59^.

### 1KGP: estimation of SVA F_1_ insertion age

Variants present in the low-coverage 1KGP3 VCF were first recoded with REF allele as the ancestral allele. SVA F_1_ diploid genotypes were then phased onto these scaffolds using the phase_common tool from SHAPEIT5. These phased haplotypes were then used as input to Relate (v.1.2.1)^60^ with 1KGP3 genomic mask to filter low mappability regions (20140520.chr20.pilot_mask.fasta.gz), modified to include the SVA F_1_ position itself. The coalescence rates provided by Relate for each 1KGP subpopulation were used.

### Estimation of SVA F insertion age

We compared the sequence of the *ASIP* SVA F with the sequences of other SVA F elements in the GRCh38 reference, which could in theory allow estimation of insertion time based on the numbers of observed base pair differences within the Alu-like and SINE-R SVA sequence elements (chr20:34222672–34223023 and chr20:34223717–34224222 at *ASIP*) and the de novo mutation rate. We ultimately concluded that these sequence elements were insufficiently long to accumulate enough mutations to allow a precise date estimate. However, we discovered that the most closely related SVA F (chr2:169785008–169787441, matching 349 of 352 bp in the Alu-like region and 505 of 506 bp in the SINE-R region) is commonly polymorphic^61^. This suggests that, despite being fixed in modern humans and archaic hominins, the *ASIP* SVA F has probably been active relatively recently and may be a relatively younger SVA in the SVA F family (which is estimated to be ~3 million years old^62^).

### GTEx genetic and expression data

Genotyping of the SVA F_1_ insertion polymorphism was performed as above on read alignments at *ASIP* from 838 donors with WGS available in the GTEx v.8 release. Because the separation of genotype clusters was less visually clear in GTEx WGS using the criteria above, the read groups informative of the two alleles were redefined more strictly as (1) discordant read pairs in which one read aligns before the SVA F_1_ left breakpoint (POS < 34228123), the other aligns after the right breakpoint (PNEXT > 34231419), and the TLEN exceeds 2.5 kb (indicative of the presence of the major allele not containing the SVA F_1_) and (2) reads aligning at least 5 bp on each side of the right breakpoint and lacking any soft-clipping (indicative of the minor allele with the SVA F_1_). Using these criteria, samples with 0 reads with TLEN >2.5 kb were called as homozygous for the SVA F_1_ insertion, and samples with fewer than three reads overlapping the right edge were called as homozygous for no SVA F_1_ insertion.

### GTEx: eQTL associations

For gene expression association analyses, the *ASIP* transcripts per million (TPM) values for a given tissue were taken from the GTEx v.8 release (GTEx_Analysis_2017-06-05_v8_RNASeQCv1.1.9_gene_tpm.gct). As some of the alternate first exons of *ASIP* were not included in the GENCODE v.26 definitions used by GTEx for expression quantification, TPM values for each exon and intronic region were computed from RNA-seq read counts. Specifically, the read counts for each region were first determined by using RNA-seq reads filtered with samtools (v.1.15.1)^63^ view for being in proper pair (-f 0x2), not failing platform/vendor quality checks (-F 0x200), having an alignment distance ≤6, and mapping quality of 255 (following GTEx; https://gtexportal.org/home/methods) as input for bedtools (v.2.27.1)^64^ coverage with -split flag. Separately, the TPM sample-level normalization factor previously computed by GTEx and applied to all genes from a given biosample was derived from read counts (GTEx_Analysis_2017-06-05_v8_RNASeQCv1.1.9_gene_reads.gct) and TPM values (GTEx_Analysis_2017-06-05_v8_RNASeQCv1.1.9_gene_tpm.gct) for *GAPDH* (computing its length as the sum of its exon lengths; gencode.v26.GRCh38.genes.gtf), as:

$$\text{TPM scaling factor}=\,\frac{\text{read counts}}{(\text{effective gene length})(\text{TPM})}$$

We used *GAPDH* to recover this biosample-level scaling factor since *GAPDH* is highly expressed across all tissues, but the choice of gene used here has a negligible effect on TPM scaling factor estimation. TPM values for each exon or intron region were then calculated by first normalizing read counts from above by the region’s size before dividing by the derived sample scaling factor.

For both gene-level and exon/intron-level eQTL analyses, the TPM values were analyzed for association with WGS-derived biallelic SNP and indel variants (GTEx_Analysis_2017-06-05_v8_WholeGenomeSeq_838Indiv_Analysis_Freeze.SHAPEIT2_phased.MAF01.vcf.gz) as well as the SVA F_1_ insertion. Analyses were performed using linear models including all GTEx v8 covariates, and conditional analyses that additionally included SVA F_1_ insertion genotype as a covariate were also performed.

Allelic fold change (aFC) was estimated as described^65^. First, in the linear regression

$$y={\beta }_{0}+{\beta }_{\rm{g}}g+{{\boldsymbol{\beta }}}_{\mathrm{cov}}{{\bf{X}}}_{\mathrm{cov}}+\varepsilon$$

the intercept, *β*_0_, estimates the expression level of two reference alleles, and the genotype effect size, *β*_g_, estimates the difference of expression between alleles (alternate − reference), where *g* are genotypes across donors, **X**_cov_ are covariates across donors, ***β***_cov_ are the coefficients for each covariate, and *ε* is noise. A point estimate of aFC can then be found as

$$\text{aFC}=\frac{2{\beta }_{\rm{g}}}{{\beta }_{0}}+1$$

where the estimated expression from reference and alternate alleles were each constrained to be positive. CIs were estimated with the adjusted bootstrap percentile (Bca) method with 10,000 replicates as implemented in R boot (v.1.3-28) library.

### GTEx: splicing QTL associations

Because the splicing phenotypes computed by GTEx consider a subset of splicing events at each locus^34^, we quantified splice events across the region defined by the longest *ASIP* isoform. RNA-seq reads were first filtered with samtools as above, after which identification and quantification of splice junctions was performed with regtools (v.0.5.2)^66^ junctions extract -a 8 -m 50 -M 500000 -s XS. This was first run on a merged bam from all GTEx biosamples corresponding to a given tissue to identify a set of nonspurious junctions (*n* > 10 observations total). The same regtools command was then run on each individual bam file, and the abundance of each junction seen in the merged set was recorded as individual-level quantification.

The fraction of reads aberrantly splicing into the SVA F splice acceptor was measured as the number of split reads supporting the junction between exon 2 and the splice acceptor within the SVA F divided by that plus the number of split reads supporting the junction between exons 2 and 3. For sQTL association analyses, the log-fraction of observed splice junctions for samples with at least one read spliced from exon 2 (adding a pseudocount of 1 to each junction count) was analyzed for association using the same approach as the eQTL analyses.

### Splice acceptor prediction

SpliceAI^36^ (v.1.3.1) was run on the GRCh38 sequence in the region of the *ASIP* intron centered roughly on the nonpolymorphic SVA F element, chr20:34221336–34224758, such that plotted SpliceAI predictions (Fig. 4b) considered at least 1 kb of sequence context on each side.

### Polyadenylation site prediction

APARENT^37^ (v.0.1) was run using the aparent_large_lessdropout_all_libs_no_sampleweights model on a region of the *ASIP* intron centered roughly on the fall-off in RNA-seq read coverage between the nonpolymorphic SVA F element and the SVA F_1_ insertion, chr20:34224584–34228084, such that plotted predictions (Fig. 4b) considered at least 1 kb of sequence context on each side.

### Cloning of CAG SVA splicing construct

pCAGEN^39^ was a gift from Connie Cepko (Addgene plasmid no. 11160; http://n2t.net/addgene:11160; RRID:Addgene_11160). mGreenLantern was synthesized as a gBlock from Integrated DNA Technologies. This was cloned into pCAGEN at the *Eco*RI and *Not*I cut sites using standard methods to yield pCAG-mGL. The human fixed SVA F was amplified from genomic DNA from 1KGP individual NA12878 using primers SVA_F and SVA_R. This amplicon then had pCAGEN-derived sequences added for Gibson assembly (New England Biolabs, cat. no. E2621S) by nested PCR with primers SVA_CAG_F and SVA_CAG_R to yield pCAG-mGL_SVA. All primer sequences can be found in Supplementary Table 1.

### Expression of CAG SVA construct, identification of splice site and reverse transcription digital droplet PCR

Plasmid pCAG-mGL_SVA was transfected into HEK293T cells (Takara Bio, cat. no. 632180) with Lipofectamine 3000 (Thermo Fisher Scientific, cat. no. L3000001) in six wells of two separate plates for a total of 12 replicates. Cells were given 24 h to express the construct before RNA was collected using Qiagen RNeasy columns (Qiagen, cat. no. 74104). To determine the exact location of the introduced splice junction, RNA was first converted to cDNA with oligo dT primers before amplifying the region spanning the expected junction between chicken beta-actin exon and SVA with CAG_bactin_fwd and CAG_SVA_rev primers. The amplicon matched the expected size (88 bp) and was Sanger sequenced with the CAG_SVA_rev primer.

To measure the relative amount of splicing into the inserted SVA F sequence, two ddPCR assays were designed: The first assay measures normal splicing between chicken beta-actin and rabbit beta-globin exons using HEX-labeled probe CAG_mGL_HEX with primers CAG_bactin_fwd and CAG_mGL_rev. The second assay measures splicing from chicken beta-actin exon to acceptor in SVA F using FAM-labeled probe CAG_SVA_FAM with primers CAG_bactin_fwd and CAG_SVA_rev. RNA was used as input with Bio-Rad One-Step RT-ddPCR Advanced Kit for Probes (Bio-Rad, cat. no. 1864022), with the optimal concentration of RNA input first identified by dilution series. Estimated Poisson-corrected concentrations of splicing into the SVA F were normalized by the sum of concentrations seen for both assays to yield an estimate of fraction spliced into the SVA F using QuantaSoft software (v.1.7). All primer and probe sequences can be found in Supplementary Table 1.

### Reporting summary

Further information on research design is available in the Nature Portfolio Reporting Summary linked to this article.

## Online content

Any methods, additional references, Nature Portfolio reporting summaries, source data, extended data, supplementary information, acknowledgements, peer review information; details of author contributions and competing interests; and statements of data and code availability are available at 10.1038/s41588-024-01841-4.

### Supplementary information

Supplementary Information Supplementary Figs. 1 and 2 and Table 1.

Reporting Summary

Peer Review File

## Data Availability

The following data resources are available by application: UKB (http://www.ukbiobank.ac.uk/) and GTEx (https://gtexportal.org/ under dbGaP accession no. phs000424.v9.p2). The following data resources are publicly available: 1KGP 30× coverage (https://www.internationalgenome.org/data-portal/data-collection/30x-grch38) and HGSVC2 (https://www.internationalgenome.org/data-portal/data-collection/hgsvc2).

## Code availability

The following publicly available software resources were used: BLAST (v.2.12.0, https://ftp.ncbi.nlm.nih.gov/blast/executables/blast+/2.12.0/), FlexiDot (v.1.06, https://github.com/molbio-dresden/flexidot), bcftools (v.1.14, http://www.htslib.org/), samtools (v.1.15.1, http://www.htslib.org/), plink (v.1.90b6.26 and v.2.00a3.7, https://www.cog-genomics.org/plink/), BOLT-LMM (v.2.4.1, https://alkesgroup.broadinstitute.org/BOLT-LMM/), susieR (v.0.12.35, https://stephenslab.github.io/susieR/), qqman (v.0.1.8), https://cran.r-project.org/web/packages/qqman/index.html), SHAPEIT5 (v.5.1.1, https://odelaneau.github.io/shapeit5/), rehh (v.3.2.2, https://cran.r-project.org/web/packages/rehh/index.html), regtools (v.0.5.2, https://regtools.readthedocs.io/en/latest/), bedtools (v.2.27.1, https://bedtools.readthedocs.io/en/latest/), SpliceAI (v.1.3.1, https://github.com/Illumina/SpliceAI), APARENT (v.0.1, https://apa.cs.washington.edu/) and Relate (v.1.2.1, https://myersgroup.github.io/relate/index.html). The following proprietary software resources were used: QuantaSoft (v.1.7, https://www.bio-rad.com/en-us/life-science/digital-pcr/qx200-droplet-digital-pcr-system/quantasoft-software-regulatory-edition). Custom code used to generate results in this study is available via Zenodo at 10.5281/zenodo.10407629 (ref. ^67^).
