# Supplementary material for: A sequence of SVA retrotransposon insertions in ASIP shaped human pigmentation
Source: Nat Genet. 2024 Jul 24;56(8):1583–91. doi: 10.1038/s41588-024-01841-4 (PMC11319198; doi:10.1038/s41588-024-01841-4)
Supplement: Supplementary file 1 — Supplementary Figs. 1 and 2 and Table 1. [file 41588_2024_1841_MOESM1_ESM.pdf]

nature genetics

Supplementary information https://doi.org/10.1038/s41588-024-01841-4

A sequence of SVA retrotransposon insertions in *ASIP* shaped human pigmentation

In the format provided by the authors and unedited

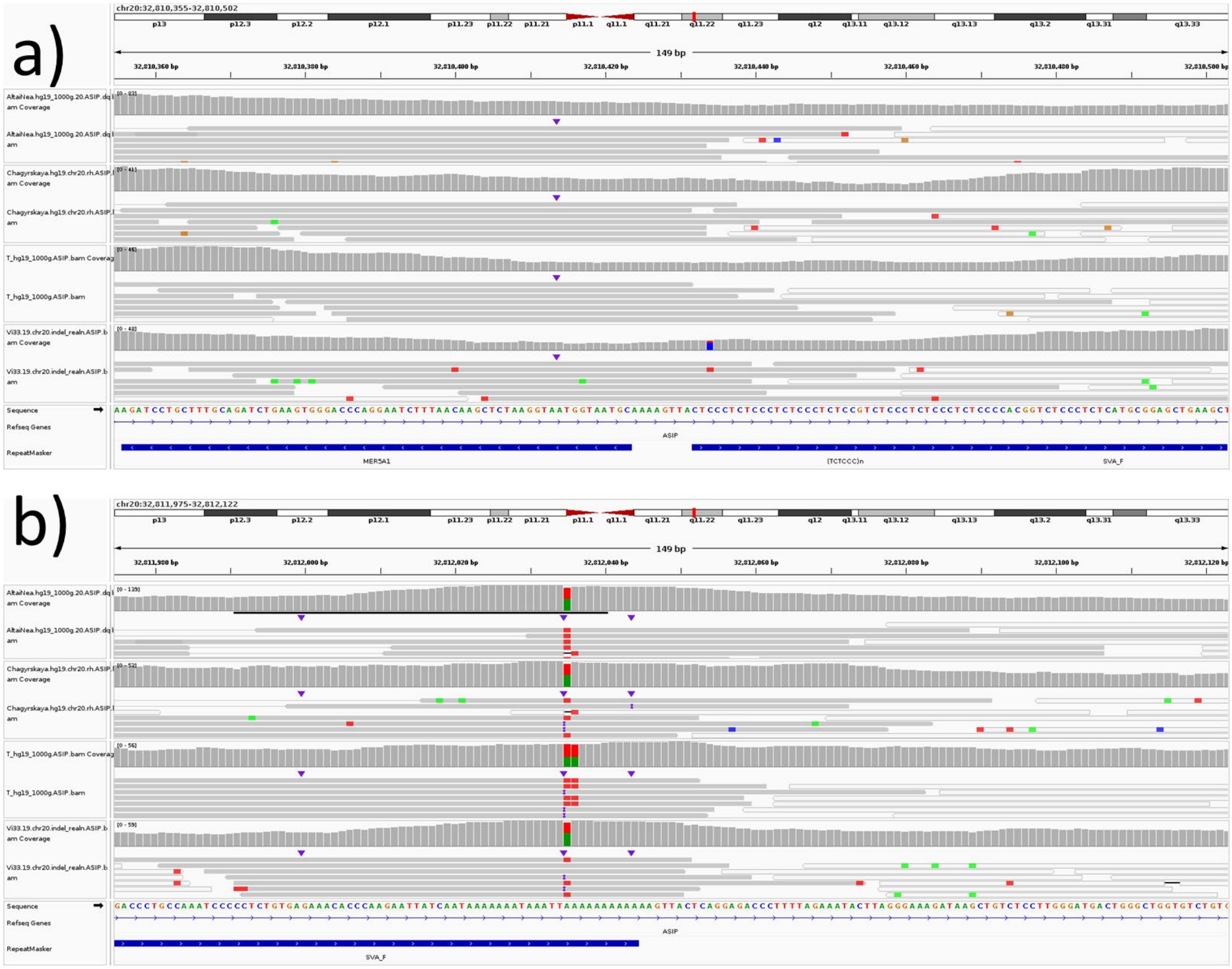

Supplementary Figure 1. High coverage Neanderthal and Denisovan alignments at SVA F breakpoints.

Human genome reference alignments (hg19) from three Neanderthal and one Denisovan high coverage sequencing libraries[40–43] have many reads that support the SVA F insertion at both the right (a) and left (b) breakpoint of the non-polymorphic SVA F. The RepeatMasker track on the bottom indicates the start and end of the SVA F; in panel (a), the 5’ end of the SVA element begins at the (TCTCCC)n hexamer repeat.

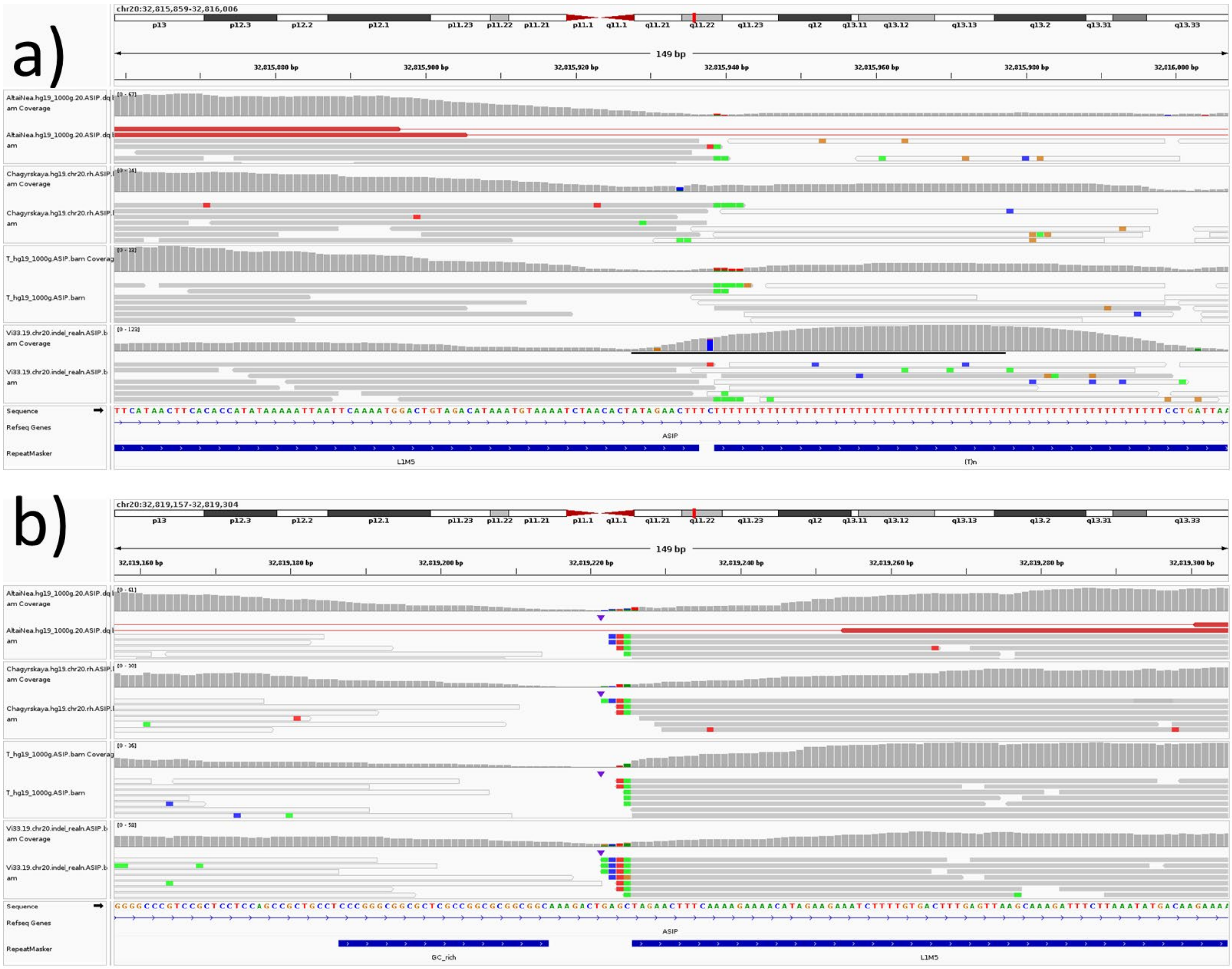

Supplementary Figure 2. High coverage Neanderthal and Denisovan alignments at SVA F_1 breakpoints.

Human genome reference alignments (hg19) from three Neanderthal and one Denisovan high coverage sequencing libraries[40–43] lack reads supporting the polymorphic SVA F_1 (as expected given its recent emergence on a European-ancestry haplotype) at either the right (a) or left (b) breakpoint of the SVA F_1. The RepeatMasker track on the bottom indicates the start and end of the SVA F_1, which inserted into an older L1M5 element. The 5' end of the SVA F_1 element (which inserted on the reverse strand) begins with the GC-rich *MAST2* exon 1 sequence (panel (b)), and the SVA F_1 finishes with a poly-A sequence on its 3' end (poly-T in panel (a)).

Name	Sequence
SVA_F	CCAGGAATCTTTAACAAGCTC
SVA_R	GAGACAGCTTATCTTTCCCTAA
SVA_CAG_F	GGTTCGGCTTCTGGCGTGTGACCGGCGGCTGTAATGGTAATGCAAAAGTTAC
SVA_CAG_R	AAGAAGGCATGAACATGGTTAGCAGAGGCTCTAGACCTAAGTATTTCTAAAAGGGTC
CAG_bactin_fwd	CTCTGACTGACCGCGTTAC
CAG_SVA_rev	GTCTCCACCAAAACCAGTCA
CAG_mGL_rev	TCCAGCTCGACCAGGATG
CAG_SVA_FAM	/56-FAM/AGGCACGCG/ZEN/CCGCCA/3IABkFQ/
CAG_mGL_HEX	/5HEX/CGAGGAGCT/ZEN/GTTCACCGGG/3IABkFQ/

Supplementary Table 1. Primer and probe sequences for cloning SVA F sequence and rt-ddPCR assays measuring aberrant splicing.
