# Supplementary material for: A sequence of SVA retrotransposon insertions in ASIP shaped human pigmentation
Source: Nat Genet. 2024 Jul 24;56(8):1583–91. doi: 10.1038/s41588-024-01841-4 (PMC11319198; doi:10.1038/s41588-024-01841-4)
Supplement: Supplementary file 2 — Reporting Summary [file 41588_2024_1841_MOESM2_ESM.pdf]

nature portfolio

Corresponding author(s): Nolan Kamitaki

Last updated by author(s): Jun 19, 2024

Reporting Summary

Nature Portfolio wishes to improve the reproducibility of the work that we publish. This form provides structure for consistency and transparency in reporting. For further information on Nature Portfolio policies, see our Editorial Policies and the Editorial Policy Checklist.

Statistics

For all statistical analyses, confirm that the following items are present in the figure legend, table legend, main text, or Methods section.

n/a	Confirmed	
☐	☒	The exact sample size (*n*) for each experimental group/condition, given as a discrete number and unit of measurement
☒	☐	A statement on whether measurements were taken from distinct samples or whether the same sample was measured repeatedly
☐	☒	The statistical test(s) used AND whether they are one- or two-sided<br>*Only common tests should be described solely by name; describe more complex techniques in the Methods section.*
☐	☒	A description of all covariates tested
☒	☐	A description of any assumptions or corrections, such as tests of normality and adjustment for multiple comparisons
☐	☒	A full description of the statistical parameters including central tendency (e.g. means) or other basic estimates (e.g. regression coefficient) AND variation (e.g. standard deviation) or associated estimates of uncertainty (e.g. confidence intervals)
☐	☒	For null hypothesis testing, the test statistic (e.g. F, t, r) with confidence intervals, effect sizes, degrees of freedom and P value noted<br>*Give P values as exact values whenever suitable.*
☒	☐	For Bayesian analysis, information on the choice of priors and Markov chain Monte Carlo settings
☒	☐	For hierarchical and complex designs, identification of the appropriate level for tests and full reporting of outcomes
☐	☒	Estimates of effect sizes (e.g. Cohen's *d*, Pearson's *r*), indicating how they were calculated

Our web collection on statistics for biologists contains articles on many of the points above.

Software and code

Policy information about availability of computer code

Data collection	QuantaSoft (v1.7) as associated with QX200 droplet reader was used to collect fluorescence measurements for digital droplet PCR.
Data analysis	The following publicly available software resources were used: BLAST (v2.12.0, https://ftp.ncbi.nlm.nih.gov/blast/executables/blast+/2.12.0/), FlexiDot (v1.06, https://github.com/molbio-dresden/flexidot), bcftools (v1.14, http://www.htslib.org/), samtools (v1.15.1, http://www.htslib.org/), plink (v1.90b6.26 and v2.00a3.7, https://www.cog-genomics.org/plink/), BOLT-LMM (v2.4.1, https://alkesgroup.broadinstitute.org/BOLT-LMM/), susieR (v0.12.35, https://stephenslab.github.io/susieR/), qqman (v0.1.8), https://cran.r-project.org/web/packages/qqman/index.html), SHAPEIT5 (v5.1.1, https://odelaneau.github.io/shapeit5/), rehh (v3.2.2, https://cran.r-project.org/web/packages/rehh/index.html), regtools (v0.5.2, https://regtools.readthedocs.io/en/latest/), bedtools (v2.27.1, https://bedtools.readthedocs.io/en/latest/), SpliceAI (v1.3.1, https://github.com/Illumina/SpliceAI), APARENT (v0.1, https://apa.cs.washington.edu/), and Relate (v1.2.1, https://myersgroup.github.io/relate/index.html). Custom code used to generate results in this study has been deposited in Zenodo doi:10.5281/zenodo.10407629.

For manuscripts utilizing custom algorithms or software that are central to the research but not yet described in published literature, software must be made available to editors and reviewers. We strongly encourage code deposition in a community repository (e.g. GitHub). See the Nature Portfolio guidelines for submitting code & software for further information.

Data

Policy information about availability of data

All manuscripts must include a data availability statement. This statement should provide the following information, where applicable:

- Accession codes, unique identifiers, or web links for publicly available datasets
- A description of any restrictions on data availability
- For clinical datasets or third party data, please ensure that the statement adheres to our policy

The following data resources are available by application: UK Biobank (http://www.ukbiobank.ac.uk/) and Genotype-Tissue Expression (GTEx) project (https://gtexportal.org/, under dbGaP accession number phs000424.v9.p2). The following data resources are publicly available: 1000 Genomes Project (1KGP) 30x coverage (https://www.internationalgenome.org/data-portal/data-collection/30x-grch38) and Human Genome Structural Variation Consortium, Phase 2 (HGSVC2) (https://www.internationalgenome.org/data-portal/data-collection/hgsvc2).

Research involving human participants, their data, or biological material

Policy information about studies with human participants or human data. See also policy information about sex, gender (identity/presentation), and sexual orientation and race, ethnicity and racism.

Reporting on sex and gender	Sex was used as a covariate in several analyses, but no values directly pertaining to sex are reported.
Reporting on race, ethnicity, or other socially relevant groupings	A subset of individuals within the UK Biobank cohort that self-identified as "white" and were not outliers (>6 standard deviations) on the first 10 genetic ancestry principal components were used for phenotype associations. Populations within 1000 genomes were analyzed according to their previously published ancestry groupings. GTEx contains individuals of multiple reported races, but this information was not used in this study.
Population characteristics	For phenotype associations, age (and age squared), sex, the top 20 genetic ancestry principal components, and intake assessment center were used as covariates.<br><br>For expression and splicing analyses, all GTEx v8 covariates were used - 5 genetic ancestry principal components, sex, PCR or PCR-free WGS preparation, Illumina sequencing platform (HiSeq 2000 or HiSeq X), and any inferred PEER covariates specific for each tissue and analysis type (expression or splicing).
Recruitment	Individuals and biosamples were not obtained for this study and their recruitment is as described in prior publications (cited in current work).
Ethics oversight	Individuals and biosamples were not obtained for this study and local IRBs at each institution approved the collections and patient-consent materials, as described in the earlier papers on these cohorts (cited in current work). Datasets were used as approved for research plans as stated in applications to each: UK Biobank Resource application #40709 and project #28875 to dbGaP accession phs000424.v9.p2 (GTEx)

Note that full information on the approval of the study protocol must also be provided in the manuscript.

Field-specific reporting

Please select the one below that is the best fit for your research. If you are not sure, read the appropriate sections before making your selection.

☒ Life sciences ☐ Behavioural & social sciences ☐ Ecological, evolutionary & environmental sciences

For a reference copy of the document with all sections, see nature.com/documents/nr-reporting-summary-flat.pdf

Life sciences study design

All studies must disclose on these points even when the disclosure is negative.

Sample size	A set of 169,641 individuals in UK Biobank with WGS available were used for all analyses, with some samples having missing information for each phenotype as noted in the text. A set of 878 individuals in GTEx with WGS available were used for all analyses, with some samples having missing RNA-seq data for each tissue as noted in the text. A set of 1508 individuals in 1KGP were used to evaluate linkage disequilibrium in the region surrounding the ASIP locus in populations from three genetic ancestral backgrounds. A subset of individuals in 1KGP (n=194) were used to estimate allele genealogy. In all cases, no sample-size calculation was done to predetermine sample size and the maximum number of available samples were used. For phenotype associations (UK Biobank), we expected that the association would be sufficiently powered to allow for fine-mapping given 1) strength of association to linked variants, 2) linkage (r2=0.97), and 3) genotyping accuracy (r=0.997). For expression and splicing associations (GTEx), the number of samples was not expected to allow for the same level of fine-mapping against other genetic variants in high linkage, but were expected to be sufficient to confirm the same association pattern was observed and the lack of any secondary signal(s) that might be considered discordant with phenotype-genotype associations. For analysis of linkage disequilibrium, we expected that the number of samples would be sufficient to show ancestral population differences given the genomic range of SNPs previously seen to associate with pigmentation phenotypes in populations of European ancestry and lack of signal seen in similar association studies from other ancestries. For estimation of allele genealogy, we expected the sample size to be sufficient given the same samples were used to generate estimates for linked variants in the original publication.

For in vitro experiments to confirm splicing into the SVA acceptor and measure in vitro splicing rate, no sample-size calculation was done to predetermine sample size as the main purpose was to observe any splice events into the SVA acceptor and confirm by Sanger sequencing. In terms of necessary sample size to accurately estimate the splicing rate, it would depend, in part, on unknown biological variability in splicing rate between SVA acceptor and downstream rabbit beta-globin exon acceptor, where the latter might cause variance to differ from that observed in endogenous ASIP exon acceptor use in skin tissue. We started with n=12 biological replicates which we determined to have a small enough sample variance that no additional replicates were needed for estimating rate.

Data exclusions

Established QC metrics were used to exclude some samples, genotypes, or sequencing data for analysis as described in previously published studies (cited in the current work). Samples from individuals in UK Biobank that requested to be withdrawn at the time of analysis were excluded. ASW and ACB populations within 1000 Genomes Project were excluded from the African genetic ancestry set in generating linkage blocks at the ASIP locus to avoid selecting variants that would have excessively long linkage due to recent admixture.

Replication

For phenotype-genotype associations, the multiple pigmentation phenotypes (skin color, tanning response, hair color, all skin cancer, non-melanoma skin cancer, and melanoma) each replicated both the overall pattern of association but also fine-mapping to the SVA as the strongest associating variant on the haplotype (with the exception of melanoma only, C43). Outside of these independently measured phenotypes from the UK Biobank cohort, these associations were not replicated or performed independently in another cohort.

Likewise, the two skin-derived tissues in GTEx (sun exposed lower leg and not sun exposed suprapubic) serve as replications for expression and splicing associations, as the measurements were from independent biosamples. The in vitro splicing assay had n=12 replicates, where all attempts were successful and included in Extended Data Figure 7.

Randomization

For UK Biobank, samples were collected in batches at different assessment centers at locations across the United Kingdom and these were encoded as indicator covariates in phenotype-genotype associations. For GTEx, samples were allocated into batches with different sample preparation methods (PCR or PCR-free) and sequencing machines (HiSeq 2000 or HiSeq X) due to changing methods during the course of their collection. These were likewise encoded as indicator covariates in expression- or splicing-genotype associations. No further randomization was done as all samples were used for each analysis.

Blinding

For all computational analyses, samples were listed with a randomized ID where association of measured genotype with trait (phenotype such as skin color or relative amount of transcript splicing) was only done at the point of final statistical analysis. Blinding was not done for the in vitro splicing assay where all samples were part of a single test group.

Reporting for specific materials, systems and methods

We require information from authors about some types of materials, experimental systems and methods used in many studies. Here, indicate whether each material, system or method listed is relevant to your study. If you are not sure if a list item applies to your research, read the appropriate section before selecting a response.

Materials & experimental systems

n/a	Involved in the study
☒	☐ Antibodies
☐	☒ Eukaryotic cell lines
☒	☐ Palaeontology and archaeology
☒	☐ Animals and other organisms
☒	☐ Clinical data
☒	☐ Dual use research of concern
☒	☐ Plants

Methods

n/a	Involved in the study
☒	☐ ChIP-seq
☒	☐ Flow cytometry
☒	☐ MRI-based neuroimaging

Eukaryotic cell lines

Policy information about cell lines and Sex and Gender in Research

Cell line source(s)	Lenti-X 293T (HEK293T clone) from Takara Bio USA; Lot#: AIY00015; Cat#: 632180
Authentication	Morphological match for type and in-house verification of SV40T antigen with genotyping PCR assay. No other standard authentication methods were performed (such as STR typing).
Mycoplasma contamination	Lack of mycoplasma contamination was done by Takara Bio USA as well as by members of receiving lab (McCarroll)
Commonly misidentified lines (See ICLAC register)	None were used in this study, HEK293T is a derivative of HEK and has not been listed as commonly misidentified.
